# Intramural duodenal hematoma: clinical course and imaging findings

**DOI:** 10.1177/2058460119836256

**Published:** 2019-04-08

**Authors:** Stefan M Niehues, Timm Denecke, Christian Bassir, Bernd Hamm, Matthias Haas

**Affiliations:** Klinik für Radiologie, Charité - Universitätsmedizin Berlin, Berlin, Germany

**Keywords:** Abdomen/GI, computed tomography, ultrasound, small bowel, adults and pediatrics, hemorrhage

## Abstract

**Background:**

Intramural duodenal hematoma is a rare condition. Different imaging modalities are at hand for diagnosis.

**Purpose:**

To identify patients with intramural duodenal hematoma and report imaging findings and clinical courses.

**Material and Methods:**

Typical imaging patterns using ultrasound, computed tomography, and magnetic resonance imaging were carried out on 10 patients.

**Results:**

The mean patient age was 7.5 years. The average disease duration was 13 months. Clinical signs of improvement were observed within 16 days. Residues were still detectable at long-term follow-up.

**Conclusion:**

For patients with intramural duodenal wall hematoma, diagnosis should be considered early. Typical imaging findings should be known to ensure optimal treatment.

## Introduction

Intramural hematomas of the vessel walls are common (1,2), but they can also occur in the intestinal wall anywhere in the gastrointestinal tract (3–7). Here, clinical signs and symptoms are remarkably different. Medical textbooks and scientific articles mention intramural duodenal hemorrhage as a rare entity. Systematic data are scarce with case reports dominating. The first case was described as early as 1838 (8) and the first source in the PubMed database accessing MEDLINE database (http://www.ncbi.nlm.nih.gov/pubmed/) is from 1952 (9). The first image of intramural duodenal hematoma appeared in an article published in 1948 (10). Most patients with intramural hematoma reported in the literature are aged <30 years (11,12).

An intramural duodenal hematoma not only has significant dietary consequences for affected patients but may also lead to potentially fatal complications. Therefore, we conducted a study to assess the frequency of intramural duodenal hematoma over a period of 20 months and to make an attempt at systematically describing its full clinical course and imaging features on different imaging modalities.

## Material and Methods

The retrospective study was approved by the local ethical committee (EA4/108/13) in accordance with the ethical standards of the World Medical Association. The inclusion period was one year. Due to protracted disease courses the total study period lasted four years, necessary to complete follow-up of all patients. During the inclusion period of one year, 10 patients diagnosed with an intramural duodenal hematoma were identified using a radiology information system (RIS) query, scanning for the words “duodenal,” “duodenum,” “wall,” and “hematoma.”

The patients had a median age of 7.5 years (age range = 3–55 years; six male patients, four female patients). Depending on the patients’ age, the primary imaging modalities used for diagnosis were ultrasound (US) or cross-sectional imaging modalities such as computed tomography (CT) and magnetic resonance imaging (MRI). All images had adequate diagnostic quality to establish the diagnosis. In two cases, the diagnosis was confirmed by endoscopy. In all patients, the underlying pathomechanism of intramural duodenal hematoma was identified. We systematically analyzed all available radiologic data to identify typical features and imaging patterns for each modality used.

## Results

Over the study period, we identified 10 patients with intramural hematoma. Patient demographics and further clinical data including diagnostic procedures are summarized in Table 1.

**Table 1.** Overview of study population: patient characteristics, imaging modalities, and clinical course.

Patient no.	Sex	Age (years)	Etiology	Anticoagulation/Coagulopathy	Hematoma size (cm)	Imaging modality	Course	Duration of disease (months)	Complications
									
1	F	5	After EGD	–	8 × 3 × 3	US/CT	Clinical improvement after 19 days	26	–
2	M	7	Spontaneously after orchidopexy	+	10 × 3 × 3	US	Clinical improvement after 13 days	6	Ileus
3	M	6	Blunt abdominal trauma	–	3.5 × 4.8 × 10.5	US	No more stenosis after 21 days	1	Pancreatic edema
4	F	19	Severe anemia	–	7 × 16 × 9.5	US/CT	Residual hematoma visible after 9 months	45	Pancreatitis and hemorrhage
5	M	37	Arrosion bleeding due to stent in hepatocholedochal duct	–	13 × 5 × 10.6	CT	Unchanged status for 21 days	No further follow-up available	Exudative pancreatitis
6	M	55	Idiopathic, aortic valve replacement	+	10 × 4 × 6	CT	Unchanged status within first 30 days	29	Transition to chronic pancreatitis
7	M	20	After deep duodenal biopsy and dialysis	+	13 × 5.5 × 5	CT/MRI	Hemorrhage, pancreatitis, organ failure	(^2^)	Death
8	M	3	Hemophilia	+	2.1 × 2.6 × 5.1	US	No improvement within the first 7 months	11	–
9	F	8	After EGD and tissue sampling	–	20 × 4 × 3	US	Return to normal lumen size after 12 days	1	–
10	F	7	After EGD and tissue sampling	–	3.5 × 4 × 5	US	No improvement within the first 14 days	1	–

Duration of disease: Maximum available follow-up until complete recovery from illness.

EGD, esophagoduodenogastroscopy.

## Clinical presentation and course

All patients presented with unspecific symptoms including abdominal pain, nausea, and vomiting. Five patients had hematemesis. Six patients had a history of abdominal trauma. In the other four patients, no acute event causing intramural hematoma could be identified. However, all four were in a hypocoagulatory state, either induced by anticoagulatory treatment or due to a coagulopathy (Table 1).

Clinical and imaging signs of improvement were seen after a mean of 16 days (range = 12–21 days). Mean disease duration until complete resolution of symptoms and disappearance of relevant imaging findings was 13 months (range = 1–45 months). Three patients had delayed recovery with persistent symptoms and signs of duodenal wall fibrosis. In one case, residual findings were still present after 45 months. One patient died.

Maximum duodenal wall diameters were in the range of 2.1–20.8 cm. Approximated duodenal wall hematoma volumes were in the range of 28–1064 mL, resulting in maximum diameters of 2.1–20.8 cm.

### Detailed report illustrating a case with a usual course

**Fig. 1.**
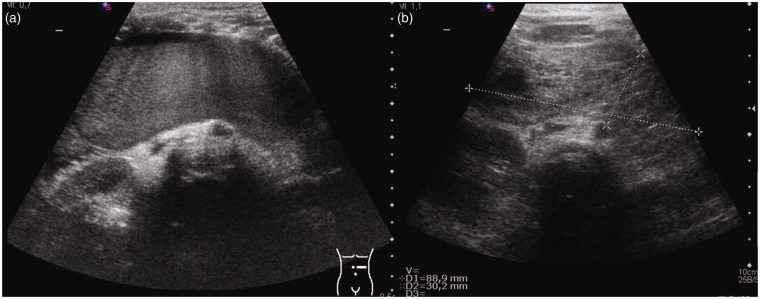
US of a five-year-old girl with a duodenal wall hematoma: (a) Initial finding with an echogenic mass which presents smaller and more cystic at follow-up (b).

**Fig. 2.**
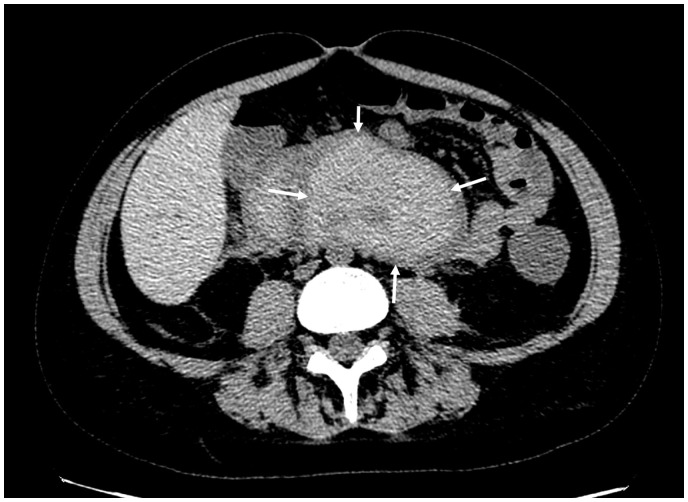
Axial contrast-enhanced CT of the pars horizontalis duodeni showing the duodenal wall hematoma (arrows) with a density of 50–60 HU.

A six-year-old boy ran into a broomstick while playing. Two days later, he was presented to the emergency room with decreasing consciousness. US revealed free abdominal fluid and a duodenal wall hematoma (Fig. 1). This was confirmed by a CT scan performed to rule out aortic injury (Fig. 2). Further follow-up included repeated US examinations, which documented a steady decrease in hematoma size with increasing liquefaction. The patient’s condition improved under fasting and parental nutrition. Twenty days after the event, no functional restriction remained, oral nutrition was tolerated, and the patient could be discharged home.

### Detailed report illustrating a worst-case scenario

**Fig. 3.**
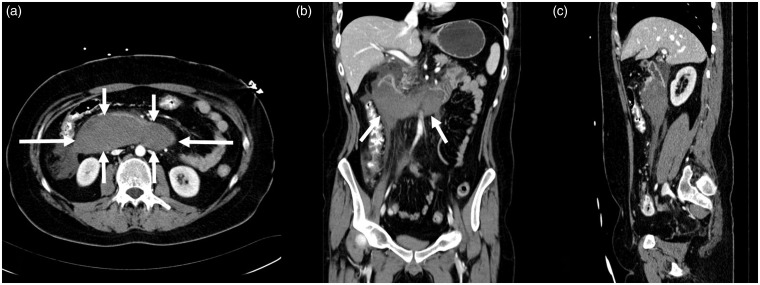
A 20-year-old man with an acute duodenal wall hematoma. Contrast-enhanced CT scan acquired at primary diagnosis in (a) axial, (b) coronal, and (c) sagittal planes demonstrates the extent of the hematoma (arrows).

**Fig. 4.**
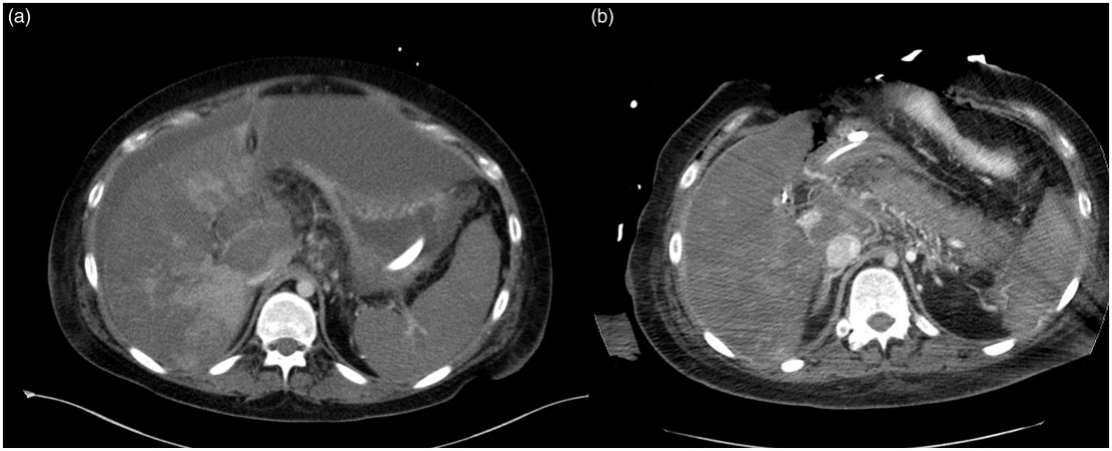
(a) Axial CT images showing extensive perfusion deficit of the liver tissue. (b) Postoperatively after decompression of the hematoma at the liver hilum reperfusion of the portal vein.

A 20-year-old male patient on hemodialysis due to renal failure from Goodpasture syndrome presented for shunt revision. The patient had no anticoagulative therapy. In the course of treatment, he developed nausea, vomiting, and diarrhea. Malabsorption was suspected and endoscopic duodenal biopsy was performed. Following biopsy, a duodenal hematoma was suspected on CT scans (Fig. 3). Endoscopy confirmed the diagnosis and showed no evidence of pancreatic duct occlusion. Still, the patient’s condition deteriorated and he developed pancreatic head pancreatitis. At the same time, CT scans showed progressive intramural hematoma. Laboratory parameters showed anemia. Pancreatitis was associated with formation of an aneurysm of the pancreaticoduodenal arterial segment. Coiling was performed, but arrosion bleeding occurred despite this measure. The intramural hemorrhage increased markedly in size, finally compressing hepatic vessels. Additionally, pancreatitis became more severe, and the patient became septic. He showed signs of beginning hepatic failure in the presence of markedly disturbed hepatic perfusion due to compression (Fig. 4a). There was no Doppler sonographically detectable flow in the portal vein (on the basis of morphologic imaging features) and markedly reduced flow in the hepatic artery. Because of the patient’s co-morbidities and his septic condition, a liver transplant was not considered promising and palliative surgery was opted for (Fig. 4b). Despite this operation, liver function did not recover. The patient died 39 days after the initial biopsy due to hepatorenal failure and intractable lactic acidosis. Pathological work-up confirmed pancreatitis and an extra- and intramural hematoma of the duodenal wall, indicating that the patient suffered from a perforated intramural hematoma.

## Systematic analysis of patterns of imaging findings

### Ultrasound

US typically reveals an echogenic mass, along the duodenal convexity, which initially has a uniform appearance (Fig. 1a). Small intramural hematoma may present as intestinal wall thickening. Yet, there may be complete obstruction of the duodenal lumen. Over time, as blood is reabsorbed, there is increasing formation of hypoechoic cystic lesions or echolucent liquid portions, while the blood volume may decrease (Fig. 1b). The late stage may be characterized by persistent echogenic focal thickening of the intestinal wall representing fibrotic scar tissue.

### CT

**Fig. 5.**
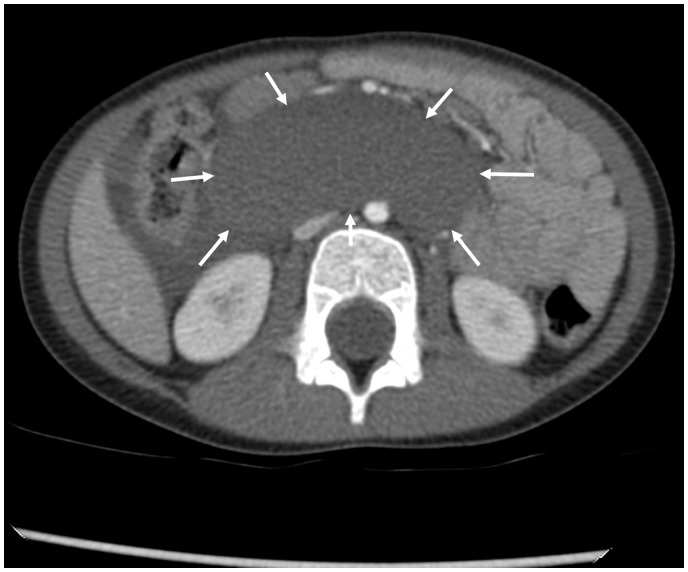
Axial low-dose contrast-enhanced CT at follow up: mixed hypo- and hyperdense mass reflecting incomplete resorption with residual clotted hematoma (arrows).

**Fig. 6.**
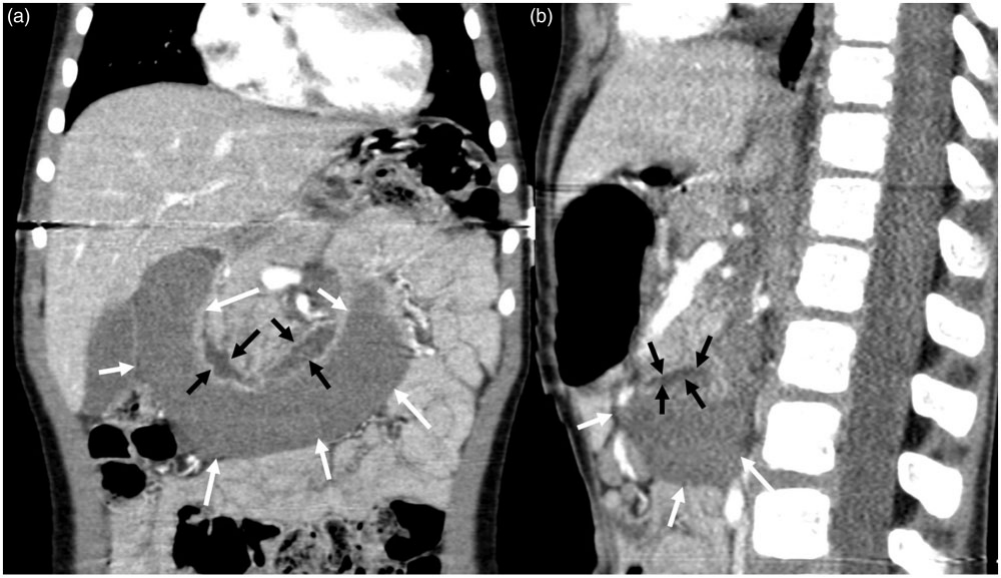
(a) Coronal and (b) sagittal CECT showing the complete extent of the hematoma (white arrows). Residual lumen of the duodenum (black arrows) seen best with additional oral contrast.

The characteristic CT finding is a mass that tends to be localized in the duodenal C displaying homogeneous density at 50–60 HU indicating coagulated blood (Fig. 5). The extent is best appreciated on coronal reconstructions (Fig. 6a). A residual lumen or the site of occlusion can often be identified on sagittal reconstructions (Fig. 6b). Use of an oral contrast agent can be helpful for evaluation. As the hematoma resolves, serial examinations show a decrease in volume and attenuation, which is due to resorption. Residual clotted blood is characterized by hyperattenuation (70–90 HU; Fig. 2). Although i.v. contrast is recommended, CT without i.v. contrast may be suitable if there are no complications (13).

### MRI

**Fig. 7.**
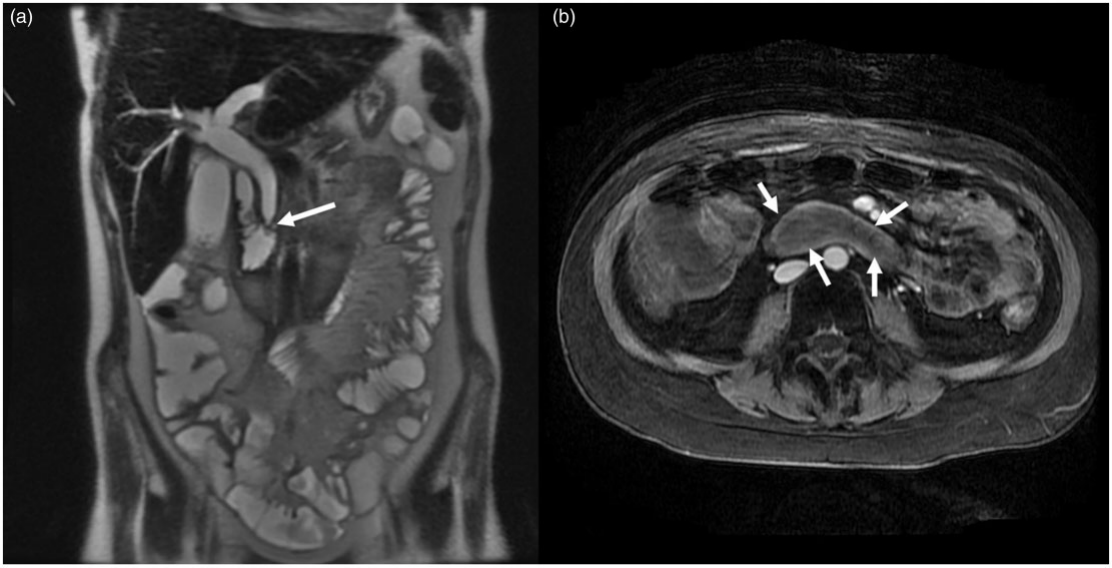
A 20-year-old male patient after deep duodenal biopsy. (a) Coronal T2W image demonstrating the hematoma size and the stenosis of the hepatic duct (arrow) as complication of the duodenal wall hematoma. (b) Axial CE T1 with fat saturation showing the extent of the duodenal wall hematoma.

MRI allows accurate evaluation of the size and extent of an intramural hematoma; as with CT, evaluation is best on coronal and axial series (Fig. 7a and b). Moreover, MRI is sensitive to early signs of bile duct dilatation and early abnormalities of the pancreatic parenchyma. As with CT, resolution of a hematoma might be identified by characteristic changes in T1 and T2 signal intensities in analogy of imaging appearance in cerebral bleedings: there, at the hyperacute stage, the oxyhemoglobin in blood results in slight hypointensity on T1-weighted (T1W) images and high signal intensity on T2-weighted (T2W) images. When oxyhemoglobin is transformed into desoxyhemoglobin, T1 signal intensity is iso- to hypointense, while T2 signal intensity is hypointense. Methemoglobin appears bright on T1W sequences; a chronic hematoma has low T1 signal intensity and high T2 signal intensity. In very late stages, both T1 and T2 signal intensities are decreased (14,15).

## Discussion

An analysis of the literature shows that the duodenum is the most common site of intramural hematoma of the gastrointestinal tract (27.5%) (11,16). The duodenum represents the widest portion of the small bowel and has no mesentery. It can be divided into four portions: a superior, a descending, a horizontal, and an ascending portion. The first, superior portion is located intraperitoneally, while the three distal portions are located retroperitoneally. The retroperitoneal attachment and the lack of a mesentery along with the proximity of especially the horizontal portion to the spine may account for vulnerability in blunt abdominal trauma (17). The suspension of the duodenojejunal junction at the ligament of Treitz is also considered a preferred site of traumatic intramural hemorrhage (18). Other sites of gastrointestinal intramural hemorrhage include the esophagus, the stomach, jejunum, ileum, and, rarely, even the colon (19,20). Published case reports on intramural duodenal hemorrhage describe several underlying causes, including a traumatic event with blunt abdominal trauma (11), duodenal ulcer (16,21), pancreatitis (22), and iatrogenic causes like duodenal biopsy or endoscopy (12,23–25).

In many published cases, intramural hematoma of the duodenum was associated with concomitant antithrombotic treatment, especially with excessive dosing (12,16,17). The authors of a survey of Swiss hospitals assumed an incidence of 1 per 2500 patients on anticoagulation treatment (26). In line with the results of our study, literature reports anticoagulant-associated bleeding to primarily occur in older individuals (mean age = 64 years (19)), while younger patients are typically reported to have a history of abdominal trauma (79% (27)).

In our population, two patients with duodenal bleeding had an endoscopy, in part with biopsy sampling. It may be assumed that this procedure involves a high risk for patients on anticoagulant treatment although none of our patients had an overdose of anticoagulation medication.

On imaging, the mural hemorrhage into the duodenal wall consistently causes narrowing or obstruction of the intestinal lumen. Most reported hemorrhages are very large; for example, Abbas et al. found the smallest diameter to be 8 cm, while the largest diameter was 23 cm (19). These data are in line with the findings in our patients, in whom hematomas had a median calculated volume of 300 mL and a median diameter of 11.6 cm.

Historically, a “coil spring” sign can be seen on upper gastrointestinal X-rays due to partial intussusception of the bowel wall distal to the hematoma (18). Today, CT is the imaging modality of choice for adults like in our cohort. It is considered useful in differentiating hematoma from perforation (28). In children, an imaging modality not involving ionizing radiation should be preferred although Hammer et al. did not find an increased cancer risk in children due to diagnostic radiation exposure (using low-dose protocols) (29,30) and many dose-optimized CT protocols are available (31). US, as in our cohort, often allows adequate primary diagnostic assessment, but is also suitable for follow-up (11). State-of-the-art MRI systems with optimized pulse sequence techniques and multichannel coils now allow rapid acquisition of high-quality data even of large volumes and are a valuable alternative to CT (32).

The initial clinical presentation of intramural duodenal hemorrhage is reported to be unspecific, comprising abdominal pain and signs of small bowel obstruction. Hematemesis is much less common (16,33). Obstruction of the papilla of Vater causes cholestasis or even pancreatitis (19,34,35). Patients with large intramural hematomas are at risk of developing anemia or even hypovolemic shock, which can also occur in the absence of perforation (16). Obstruction of duodenal passage has been reported in all documented cases.

There is wide variation in the time it takes for a duodenal hematoma to resolve. Sixty percent of the patients in our study improved within a short period, while 30% had delayed recovery with a maximum follow-up of 45 months. This might be due to continued anticoagulation therapy and, in one case, was attributable to the extremely large hematoma volume of 1064 mL. Other investigators have reported short-term improvement in 85% of cases and 15% mortality (19,25,34).

The primarily recommended treatment option is watchful waiting. In individual cases, rapid improvement may be achieved with surgery with or without duodenal wall incision (16,24,27), but results in longer hospital stays (36). In rare cases, parenteral nutrition is required. In adult patients with active bleeding or pseudoaneuryms, transarterial embolization can be effective (37).

As a limitation of our study, the numbers investigated with any one imaging modality are too small for meaningful conclusions to be drawn regarding any one of these modalities. For the same reason, we cannot definitely rule out additional typical imaging findings.

In conclusion, intramural duodenal hemorrhage is an acute condition and possible complications can be reduced by early diagnosis. Complications range from occlusion or stricture of the duodenum to pancreatitis, arrosion hemorrhage, and clinical shock. In young patients with blunt abdominal trauma and patients presenting with abdominal pain who vomit bile and blood, intramural hemorrhage should be ruled out, particularly in those on anticoagulation treatment. While older patients are examined by MRI or CT, young patients can be examined by US and may require an additional MRI or CT only if US findings are inconclusive.
